# The mediating effect of error management climate on the relationship between nursing informatics competency and information security attitude among clinical nurses: a cross-sectional study

**DOI:** 10.3389/fpubh.2026.1887079

**Published:** 2026-07-10

**Authors:** Yurui Xue, Changqi Yin, Xue Bai, Shuangdui Ji, Fuzhen Ma, Donglian Zheng, Lu Yang, Yaqin Ma, Yuyuan Zhang, Wei Zhang, Shuping Guo

**Affiliations:** 1 School of Nursing, Ningxia Medical University, Yinchuan, Ningxia, China; 2 Department of Cardiovascular Surgery, General Hospital of Ningxia Medical University, Yinchuan, Ningxia, China; 3 Department of Orthopedic Trauma, General Hospital of Ningxia Medical University, Yinchuan, Ningxia, China; 4 Department of Nursing, General Hospital of Ningxia Medical University, Yinchuan, Ningxia, China

**Keywords:** error management climate, information security attitude, mediating effect, nurses, nursing informatics competency

## Abstract

**Background:**

With the development of information technology, the issue of data leakage in hospitals has become increasingly prominent. As the primary personnel who handle sensitive data directly, the information security attitudes of nurses are a critical determinants of medical information safety. However, no studies have explored the relationships among nursing informatics competency, error management climate, and the information security attitudes of nurses. This study aimed to examine the influence of nursing informatics competency on the information security attitudes of nurses and to verify the mediating role of error management climate.

**Methods:**

This study adopted a cross-sectional design and recruited 764 clinical nurses from 10 public secondary or higher-level hospitals across 5 cities in Ningxia Hui Autonomous Region between November 2024 and March 2025 using a convenience sampling method. Data were collected using a general information questionnaire, the Nursing Informatics Competency Scale, the Error Management Climate Scale, and the Information Security Attitude Scale. Data Analyses included descriptive statistics, Spearman correlation, and mediation modeling using the PROCESS Macro in SPSS 27.0.

**Results:**

The results of the correlation analysis showed that nursing informatics competency was positively correlated with information security attitude (*r* = 0.582, *p* < 0.001) and positively correlated with error management climate (*r* = 0.497, *p* < 0.001). Error management climate was significantly positively correlated with information security attitude (*r* = 0.635, *p* < 0.001). The results of the mediation analysis showed that the total effect of nursing informatics competency on clinical nurses’ information security attitude was 0.495, of which the direct effect was 0.286, accounting for 57.78% of the total effect, and the effect of error management climate as a mediating variable was 0.209, accounting for 42.22% of the total effect.

**Conclusion:**

Information security attitude among clinical nurses needs to be further improved, and nursing informatics competency is a positive predictor of information security attitude. In addition, error management climate plays a partial mediating role in the relationship between nursing informatics competency and information security attitude among nurses. These findings suggest that nursing managers should pay attention to and improve the nursing informatics competency of clinical nurses and create a positive error management climate to enhance information security attitude and behavior, thereby ensuring patient information security.

## Background

With the rapid development of information technology, the global healthcare industry is undergoing a profound digital transformation. To follow this trend, the China Nursing Development Plan (2021–2025) (1) clearly requires focused strengthening of nursing informatization construction to develop smart hospitals. Driven by this, the hospital information system (HIS) has been widely applied in clinical nursing work, greatly improving the efficiency and quality of nursing care. However, while digital transformation brings convenience, it is also accompanied by severe information security challenges (2). According to the Verizon Data Breach Investigations Report (3), the healthcare industry has become one of the sectors with the most frequent data breach incidents, among which about one-third of security events can be attributed to unintentional human errors by internal personnel (4). Nursing information security is an important component of medical information security. Nursing work often involves large amounts of sensitive patient information, including personal identifiers and medical records, and so on. Once such information is leaked, tampered with, or lost, it will not only infringe on patient privacy, but may also trigger medical disputes and even affect the reputation and normal operation of hospitals (5). Studies have shown that most medical information security problems are related to the weak information security awareness and improper behaviors of healthcare workers (6, 7). Attitudes are closely related to behaviors, and research has showed that coping behaviors among nurses can be predicted by assessing them information security attitudes (8, 9). As the main group directly contacting and collecting patient information in frontline clinical work, nurses undertake key responsibilities in the prevention and management of information security incidents (10). Their attitudes toward information security and their actual behavioral performance will directly determine the level of medical information security. At present, information security attitudes among clinical nurses are generally insufficient to fully cope with the needs of an increasingly complex medical information environment (11, 12), with obvious shortcomings especially in information literacy and security practices (13, 14).

Nursing informatics competency, as a core competency that aligns with the demands of clinical positions, is the comprehensive ability of nurses in terms of knowledge, skills, and attitudes displayed when handling various information activities in nursing work (15). This competency promotes effective nursing informatics behaviors, which in turn contribute to the protection of patient information security and the enhancement of nursing care quality (16, 17). Nursing informatics competency has been identified by the American Association of Colleges of Nursing as one of the fundamental proficiency requirements essential in the training of clinical nurses (18). The knowledge-Attitude-Practice (KAP) theory holds that knowledge is the cognitive basis for forming a positive attitude (19). In the field of nursing information, nursing informatics competency of clinical nurses, as the core embodiment of their professional knowledge and skills, directly constitute the cognitive basis of their information security attitude. Studies have shown that the higher the level of nursing informatics competency among clinical nurses, the better them information security attitude (20, 21). Nurses with higher levels of nursing informatics competency usually possess stronger abilities in information acquisition, processing, and application, and this competency promotes a deeper understanding of the importance of information technology and information security management, thereby forming a more positive information security attitude (12).

However, although nursing informatics competency is an important foundation influencing information security attitudes, clinical practice has found that merely improving nurses’ information skills does not necessarily translate effectively into a positive information security attitude. The reason is that traditional knowledge and skills training focuses only on strengthening skills at the “surface level of behavior,” while neglecting situational factors as an internal driving force in the process of transforming individual cognition into attitudes and behaviors (22, 23). Due to the complexity and uncertainty of clinical nursing work, the occurrence of errors is difficult to completely avoid. Therefore, nurses need to reduce potential risks by actively identifying, reporting, and analyzing their own errors. Error management climate refers to the cultural atmosphere formed within an organization regarding attitudes toward errors, cognition of errors, and ways of handling errors (24). It not only reflects the organization’s fundamental attitude and behavioral style toward errors, but also more profoundly influences employees’ perception of risk and them tendencies in behavioral decision-making (25). Studies have shown that error management climate is directly related to attitudes and behaviors in nursing work (26, 27). At present, due to a lack of professional self-awareness and cognitive level, some clinical nurses show insufficient error management in their actual work. This phenomenon not only reduces the work efficiency and quality of the entire nursing team, but at the level of information security it also means that risks such as data leakage and non-compliant operations are concealed and accumulated, which will ultimately systematically break through the defense line of patient information security and restrict the continuous improvement and healthy development of the hospital nursing service quality.

Previous studies have mostly focused on exploring the current status of nursing informatics competency and information security attitudes, or on analyzing the correlations of nursing informatics competency and error management climate with information security attitudes. Systematic research on the interactions among nursing informatics competency, error management climate, and information security attitudes is still insufficient. Therefore, this study introduces error management climate as a mediating variable, aiming to explore the pathway through which nursing informatics competency affects information security attitudes among clinical nurses, and to verify the mediating role of error management climate, to provide a reference for optimizing error management mechanisms and formulating intervention measures for information security management strategies.

### Theoretical framework

Social Cognitive Theory (SCT) was proposed by Albert Bandura (28). Its core concept of triadic reciprocal determinism emphasizes the dynamic interaction among the individual, behavior, and environment. In this interactive process, individual cognition (such as knowledge and competence) and environmental factors (such as organizational climate and leadership support) jointly determine outcome expectations and self-efficacy, thereby shaping attitudes and behaviors. According to the triadic reciprocal determinism of SCT, an explanatory pathway with “competence environment-attitude” as the core was constructed. In this study, nursing informatics competency represented the individual cognitive and skill dimension of nurses in information processing and was the key basis for shaping their self-efficacy regarding information security; error management climate constituted the key organizational environmental dimension influencing nurses’ cognitive processes, and an open, non-punitive climate can positively change nurses’ outcome expectations regarding reporting information security errors. This was used to verify the effects of these two factors on information security attitudes among clinical nurses.

Integrating insights from theoretical and empirical research, we proposed the following four hypotheses:

*H1*: Nursing informatics competency has a positive predictive effect on information security attitudes of clinical nurses.

*H2*: Error management climate has a positive predictive effect on information security attitudes of clinical nurses.

*H3*: Nursing informatics competency has a positive predictive effect on error management climate.

*H4*: Error management climate mediates the relationship between nursing informatics competency and information security attitudes of clinical nurses.

**Figure 1:**
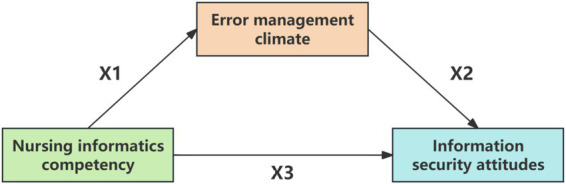
Theoretical model and hypotheses.

The hypothetical model is shown in Figure 1.

## Methods

### Study design

This study adopted a cross-sectional study design.

### Participants and sample

Use researchers recruited 764 clinical nurses through convenience sampling from 10 secondary and tertiary public hospitals across five cities in the Ningxia Hui Autonomous Region (Yinchuan, Shizuishan, Guyuan, Wuzhong, and Zhongwei) during the period from November 2024 to March 2025. The inclusion criteria were: (a) having obtained a nurse practice qualification certificate; (b) agreeing to and voluntarily participating in this survey. The exclusion criteria were: (a) nurses who came to the hospital for further study or training during the survey period; (b) nurses who were not on duty during the survey period, such as those on maternity leave, sick leave, or studying outside the hospital.

To determine the appropriate sample size, an *a priori* Monte Carlo power analysis was performed based on pilot data drawn from the original investigation (29). This analysis aimed to identify the number of participants needed to achieve a statistical power of 0.80. The parameters specified included a maximum sample size of 1,000, 1,000 Monte Carlo repetitions, a 95% confidence interval, and default software configurations (e.g., random seed fixed at 1234). The resulting estimates indicated that a minimum of 262 participants would be required to reach the desired power level. Given that the final analytic sample comprised 764 participants—substantially exceeding this threshold—the study was deemed adequately powered. Ultimately, the effective sample size of 724 cases confirmed sufficient statistical support for the proposed mediation model.

### Instruments

#### Demographic information

Based on reviewing previous literature and combining clinical practice experience. This includes items such as gender, age, marital status, educational level, hospital level, professional title, nursing level, clinical position, department, employment type, years of work, whether engaged in clinical teaching, whether participated in information security training (internal/external), whether involved in hospital informatization development, and whether information security training is conducted in the department, totaling 15 items.

#### Nursing informatics competency scale

The Nursing Informatics Competency Scale was developed by Luo et al. (30) based on systematic theoretical research and was used to assess the nursing informatics competency of clinical nursing staff. The scale includes five dimensions: nursing information awareness (4 items), computer operation skills (8 items), computer management competency (5 items), nursing information operation ability (3 items), and nursing information management ability (12 items), with a total of 32 items. A 5-point Likert scoring method is used, with 1 representing “completely inconsistent” and 5 representing “completely consistent.” The total score ranges from 32 to 160 points, and a higher score indicates a higher level of nursing informatics competency. The Cronbach’s *α* coefficient of the scale is 0.947. In this study, the Cronbach’s α coefficient of the scale was as high as 0.957.

#### Error management climate scale

The Error Management Climate Scale, originally developed by van Dyck et al. (31), was later translated and adapted into Chinese by Du et al. (32). This scale is designed to assess individuals’ perceptions of error management within their organization. It includes four dimensions: error learning (4 items), error reflection (5 items), error competence (3 items), and error communication (4 items), with a total of 16 items. A 5-point Likert scoring method is used, with 1 representing “strongly disagree” and 5 representing “strongly agree.” The total score ranges from 16 to 80 points, and a higher score indicates a better perceived error management climate in the nurse’s department. The Cronbach’s *α* coefficient of the scale is 0.924. In this study, the Cronbach’s α coefficient of the scale was 0.827.

#### Information security attitude questionnaire

The Information Security Attitude Questionnaire was developed by Kang et al. (33) and subsequently translated and culturally adapted into Chinese by Liu et al. (34). This scale is used to assess the information security attitudes of nurses toward patients data. This questionnaire includes 30 items across six dimensions: environmental control (3 items), device stability maintenance (4 items), information access restriction (4 items), system stability in work practice (8 items), enhanced professional responsibility (6 items), and continuous education and training (5 items). A 4-point scoring method is used, with 1 representing “strongly disagree” and 4 representing “strongly agree.” The total score ranges from 30 to 120 points, and a higher score indicates a higher level of nurses’ information security attitude. The Cronbach’s *α*coefficient of the scale is 0.870, and in this study the Cronbach’s α coefficient of the scale was 0.830.

#### Data collection

The survey was administered through the certified online platform “Questionnaire Star”^1^. The first page contained instructions for completion, survey guidelines, and the informed consent statement. First, the head nurse directors of the nursing departments of each hospital were contacted separately, the purpose and the significance of this study were explained, and, after obtaining their consent, the electronic questionnaire link was forwarded to nurses in each department for completion. All questionnaires were completed voluntarily. The survey was anonymous, and each IP address was allowed to submit only one response. The background system of the online questionnaire automatically monitored the response time, and the questionnaire could be submitted only after all items were completed without missing data. Two researchers jointly checked the data, and questionnaires with a completion time of <180 s or with obviously patterned or logically confused responses were considered invalid and excluded.

#### Data analysis

SPSS 27.0 and SPSS PROCESS 4.2 software were used for data processing and analysis. Measurement data that did not follow a normal distribution were described using the median and quartile (*P*_25_, *P*_75_). The Mann–Whitney U test was used for comparisons between two groups, and the Kruskal-Wallis H test was used for comparisons among multiple groups. Spearman correlation analysis was then performed to examine the correlations among nurses’ nursing informatics competency, error management climate, and information security attitude. Harman’s single-factor test was used to examine common method bias. In addition, we used the PROCESS macro (model 4) in SPSS software to analyze the mediating effect of error management climate, and the bias-corrected percentile bootstrap method was adopted for testing, with 5,000 bootstrap resamples used to calculate the 95% confidence interval. If the 95% confidence interval did not contain 0, the mediating effect was considered statistically significant. The significance level was set at *α* = 0.05.

#### Ethical considerations

This study was approved by the Ethics Committee of Ningxia Medical University (approval number: KYLL-2023-0188). All participants were informed of the purpose of the study and signed informed consent forms. The research process strictly followed the ethical principles of the Declaration of Helsinki.

## Results

### Comparison of information security attitudes among nurses with different demographic characteristics

Descriptive statistics, Mann–Whitney U test and Kruskal-Wallis H test were used to describe and compare the demographic characteristics of clinical nurses and them information security attitude scores, as shown in Table 1.

**Table 1:** Comparison of information security attitudes among nurses with different demographic characteristics (*N* = 764).

Variables	*N* (%)	Median (*P_25_, P_75_)*	*Z/H_C_*	*p*
Gender				
Male	27(3.5)	86.00 (82.00,96.00)	−1.390	0.165
Female	737 (96.5)	94.00 (82.00,99.00)		
Age (years)				
≤30	202 (26.4)	96.00 (85.75,100.00)	13.816	<0.001
31 ~ 40	373 (48.8)	94.00 (82.00,99.00)		
41 ~ 60	189 (24.7)	92.00 (80.00,98.00)		
Marital status				
Single	77 (10.1)	95.00 (85.00,99.50)	3.443	0.179
Married	671 (87.8)	94.00 (82.00,99.00)		
Other	16 (2.1)	92.00 (78.25,98.75)		
Educational level				
Junior college	139 (18.2)	93.00 (82.00,99.00)	1.744	0.418
Bachelor’s degree	619 (81.0)	94.00 (82.00,99.00)		
Postgraduate	6 (0.8)	92.50 (87.25,99.50)		
Hospital level				
Tertiary hospital	572 (74.9)	95.00 (84.00,99.00)	−4.739	<0.001
Secondary hospital	192 (25.1)	87.50 (80.00,97.00)		
Professional title				
Staff nurse	98 (12.8)	94.00 (83.00,99.00)	1.420	0.701
Senior staff nurse	282 (36.9)	93.00 (81.00,99.00)		
Nurse supervisor	319 (41.8)	95.00 (82.00,99.00)		
Deputy chief nurse or above	65 (8.5)	95.00 (86.00,98.50)		
Nursing level				
N0	67 (8.8)	88.00 (80.00,98.00)	4.074	0.396
N1	100 (13.1)	94.00 (83.00,99.00)		
N2	260 (34.0)	95.00 (82.00,99.00)		
N3	274 (35.9)	94.00 (82.00,99.00)		
N4	63 (8.2)	94.00 (83.00,98.00)		
Clinical position				
Staff nurse	575 (75.3)	94.00 (82.00,99.00)	3.025	0.220
Charge nurse	101 (13.2)	96.00 (85.00,99.00)		
Head nurse	88 (11.5)	94.50 (83.25,99.00)		
Department				
Internal medicine	308 (40.3)	95.00 (83.00,99.00)	8.226	0.313
Surgery	169 (22.1)	95.00 (81.00,99.00)		
Gynecology	45 (5.9)	95.00 (86.00,99.50)		
Pediatrics	41 (5.4)	95.00 (87.00,99.00)		
Emergency department	40 (5.2)	95.00 (84.00,99.75)		
Operating room	38 (5.0)	86.00 (80.00,97.25)		
ICU	70 (9.2)	91.00 (81.00,98.00)		
Outpatient clinic	53 (6.9)	93.00 (81.00,98.00)		
Employment type				
Contract-based	578 (75.7)	95.00 (83.00,99.00)	7.329	0.026
Permanent staff	166 (21.7)	93.00 (81.00,98.00)		
Other	20 (2.6)	89.00 (78.25,96.50)		
Years of work				
≤3	69 (9.0)	94.00 (84.00,99.00)	1.449	0.694
4 ~ 10	256 (33.5)	94.50 (82.00,99.00)		
11 ~ 14	161 (21.1)	95.00 (82.50,99.00)		
15 ~ 36	278 (36.4)	94.00 (82.00,98.00)		
Whether engaged in clinical teaching				
Yes	429 (56.2)	95.00 (83.00,99.00)	−1.289	0.197
No	335 (43.8)	93.00 (82.00,99.00)		
Whether participated in information security training (internal/external)				
Yes	678 (88.7)	95.00 (83.00,99.00)	−5.064	<0.001
No	86 (11.3)	85.00 (78.00,94.25)		
Whether involved in hospital informatization development				
Yes	552 (72.3)	95.00 (84.00,99.00)	−4.617	<0.001
No	212 (27.7)	90.00 (79.25,97.00)		
Whether information security training is conducted in the department				
Yes	688 (90.1)	95.00 (84.00,99.00)	−6.655	<0.001
No	76 (9.9)	81.00 (77.00,91.50)		

A total of 764 nurses were included in this study. The majority of participants were females (*n* = 737, 96.5%), and a larger proportion were aged 31–40 years (*n* = 373, 48.8%). In terms of educational level, most nurses held a bachelor’s degree (*n* = 619, 81.0%). Regarding professional title, 98 were nurses (12.8%), 282 were senior nurses (36.9%), 319 were nurse-in-charge (41.8%), and 65 were associate chief nurse or above (8.5%). In addition, according to the commonly used nursing grade management system in China, 67 (8.8%) were at N0 level, 100 (13.1%) at N1 level, 260 (34.0%) at N2 level, 274 (35.9%) at N3 level, and 63 (8.2%) at N4 level. More detailed data are shown in Table 1.

A univariate analysis of information security attitude was conducted among the 764 clinical nurses who participated in the survey. The results showed that there were statistically significant differences in information security attitude scores in terms of age, hospital level, employment type, whether they participated in information security training (internal/external), whether involved in hospital informatization development, and whether information security training is conducted in the department (*p* < 0.05). More detailed data are presented in Table 1.

### Descriptive results of nursing informatics competency, error management climate and information security attitude

Table 2 presents the scores of nursing informatics competency, error management climate and information security attitude among the 764 clinical nurses who participated in this study. The total score of nursing informatics competency was 127.00 (116.00, 141.00), the total score of error management climate was 64.00 (57.00, 68.00), and the total score of information security attitude was 94.00 (82.00, 99.00).

**Table 2:** Scores of nursing informatics competency, error management climate and information security attitude (*N* = 764).

Items	Total score median (*P_25_, P_75_)*	Average score median (*P_25_, P_75_)*
Nursing informatics competency	127.00 (116.00,141.00)	3.97 (3.63,4.41)
Nursing information awareness	16.00 (14.00,17.00)	4.00 (3.50,4.25)
Computer operational skills	32.00 (29.00,36.00)	4.00 (3.63,4.50)
Computer management competency	20.00 (20.00,24.00)	4.00 (4.00,4.80)
Nursing informatics operational ability	14.00 (12.00,15.00)	4.67 (4.00,5.00)
Nursing informatics management ability	47.00 (39.00,51.00)	3.92 (3.25,4.25)
Error management climate	64.00 (57.00,68.00)	4.00 (3.56,4.25)
Error learning	16.00 (15.00,17.00)	4.00 (3.75,4.25)
Error reflection	20.00 (18.00,22.00)	4.00 (3.60,4.40)
Error competence	12.00 (10.00,13.00)	4.00 (3.33,4.33)
Error communication	16.00 (14.00,17.00)	4.00 (3.50,4.25)
Information security attitude	94.00 (82.00,99.00)	3.13 (2.73,3.30)
Environmental control	9.00 (8.00,11.00)	3.00 (2.67,3.67)
Device stability maintenance	11.00 (9.00,12.00)	2.75 (2.25,3.00)
Information access restriction	11.00 (10.00,12.00)	2.75 (2.50,3.00)
System stability in work practice	25.00 (22.00,27.00)	3.13 (2.75,3.38)
Enhanced professional responsibility	19.00 (17.00,21.00)	3.17 (2.83,3.50)
Continuous education and training	15.00 (14.00,18.00)	3.00 (2.80,3.60)

### Correlation analysis of nursing informatics competency, error management climate and information security attitude

As shown in Table 3, clinical nurses’ nursing informatics competency was positively correlated with error management climate (*r* = 0.497, *p* < 0.001) and information security attitude (*r* = 0.582, *p* < 0.001). With the enhancement of nursing informatics competency, both error management climate and information security attitude increased accordingly. It is noteworthy that the correlation between error management climate and information security attitude were the strongest (*r* = 0.635, *p* < 0.001). This indicates that as the error management climate of clinical nurses increases, them information security attitude also improves significantly.

**Table 3:** Correlations among nursing informatics competency, error management climate and information security attitude (*N* = 764).

Variables	1	2	3
1. Nursing informatics competency	1		
2. Error management climate	0.497**	1	
3. Information security attitude	0.582**	0.635**	1

*** p < 0.001.

### Common method bias test

Harma’s single-factor test was used to examine common method bias. After principal component analysis was conducted on all scale items involved in the hypothesis testing, 13 common factors with eigenvalues greater than 1 were obtained, among which the first common factor explained 23.66% of the variance, which was lower than the critical threshold of 40%. This indicates that there was no serious common method bias in this study.

### Results of hierarchical multiple regression analysis

We used PROCESS Model 4 to adjust for covariates and test the mediating effect. All dependent variables, independent variables and mediating variables were standardized. To exclude the interference of demographic information on the study results, significant demographic variables (*p* < 0.05) in the univariate analysis, such as age and hospital level, were included as control variables in the hierarchical multiple models. In the hierarchical multiple linear regression analysis, nursing informatics competency was taken as the independent variable, error management climate as the mediating variable, and information security attitude as the dependent variable. As shown in Table 4, the results indicated that nursing informatics competency had a significant total effect on information security attitude (c = 0.495, SE = 0.030, *t* = 16.573, *p* < 0.001), and the initial *R^2^* was 0.360. In addition, nursing informatics competency significantly predicted error management climate (*a* = 0.433, SE = 0.032, *t* = 13.661, *p* < 0.001). When both nursing informatics competency and error management climate were entered into the model, the direct effect of nursing informatics competency on information security attitude remained significant (c’ = 0.286, SE = 0.029, *t* = 9.980, *p* < 0.001), and error management climate had a positive predictive effect on information security attitude (*b* = 0.482, SE = 0.029, *t* = 16.372, *p* < 0.001). After introducing the mediating variable, the explanatory power of the model *R^2^* increased from 0.360 to 0.527, suggesting that error management climate may play a mediating role between nursing informatics competency and information security attitude among nurses.

**Table 4:** Hierarchical multiple linear regression analysis results (*N* = 764).

Regression model		Overall fit index			Significance of regression coefficients				
Variables	Independent variables	*R*	*R^2^*	F	*β*	SE	*t*	*p*	95% CI
Error management climate		0.527	0.278	41.600					
	Nursing informatics competency				0.433	0.032	13.661	<0.001	[0.371,0.495]
	Age				−0.100	0.047	−2.136	0.033	[−0.192,-0.008]
	Hospital level				−0.108	0.072	−1.496	0.135	[−0.249,0.034]
	Employment type				−0.053	0.067	−0.792	0.429	[−0.185,0.079]
	Participation in internal/external information security training courses				0.075	0.118	0.633	0.527	[−0.157,0.306]
	Involvement in hospital informatization development				−0.197	0.077	−2.568	0.010	[−0.348,-0.047]
	Departmental provision of information security training				−0.561	0.124	−4.547	<0.001	[−0.804,-0.319]
Information security attitude		0.726	0.527	105.294					
	Nursing Informatics competency				0.286	0.029	9.980	<0.001	[0.230,0.342]
	Error Management Climate				0.482	0.029	16.372	<0.001	[0.424,0.540]
	Age				−0.064	0.038	−1.692	0.091	[−0.139,0.010]
	Hospital level				−0.231	0.058	−3.953	<0.001	[−0.346,-0.116]
	Employment type				−0.037	0.055	−0.672	0.502	[−0.144,0.070]
	Participation in internal/external information security training courses				−0.106	0.095	−1.114	0.266	[−0.294,0.081]
	Involvement in hospital informatization development				−0.045	0.063	−0.724	0.469	[−0.168,0.077]
	Departmental provision of information security training				−0.208	0.101	−2.055	0.040	[−0.407,-0.009]
Information security attitude		0.600	0.360	60.628					
	Nursing informatics competency				0.495	0.030	16.573	<0.001	[0.436,0.553]
	Age				−0.113	0.044	−2.552	0.011	[−0.199,-0.026]
	Hospital level				−0.283	0.068	−4.169	<0.001	[−0.416,-0.150]
	Employment type				−0.062	0.063	−0.984	0.326	[−0.187,0.062]
	Participation in internal/external information security training courses				−0.070	0.111	−0.634	0.526	[−0.288,0.147]
	Involvement in hospital informatization development				−0.140	0.072	−1.940	0.053	[−0.282,0.002]
	Departmental provision of information security training				−0.479	0.116	−4.117	<0.001	[−0.707,-0.251]

### The mediating effect of error management climate between nursing informatics competency and information security attitude

**Figure 2:**
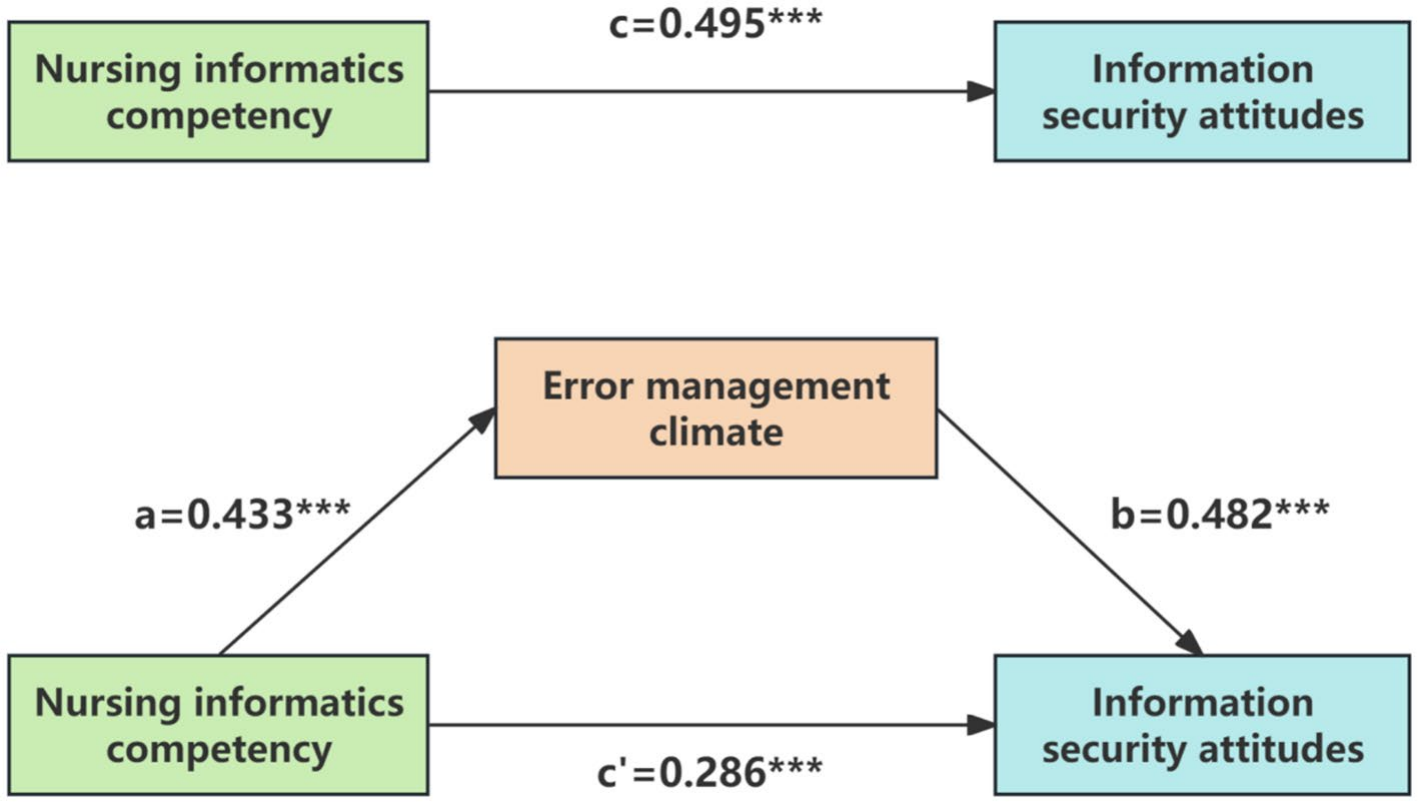
The mediating model of nurses’ error management climate in the relationship between nursing informatics competency and information security attitude, ***p < 0.001.

Model 4 in the PROCESS 4.2 macro of SPSS software was used to construct the mediation model and to examine the mediating role of error management climate between nursing informatics competency among clinical nurses and information security attitude. The bootstrap method with 5,000 resamples was applied, and a 95% confidence interval (CI) that did not include 0 was considered statistically significant (*p* < 0.05). The results showed that the 95% CI for the total effect of nursing informatics competency on information security attitude was 0.436 to 0.553, with the 95% CI for the direct effect being 0.230 to 0.342 and that for the indirect effect being 0.170 to 0.250, all of which did not include 0, indicating that error management climate exerted a partial mediating effect between nursing informatics competency among clinical nurses and information security attitude. The total effect of nursing informatics competency on information security attitude was 0.495, of which the direct effect of 0.286 accounted for 57.78% of the total effect, and the mediating effect of error management climate of 0.209 accounted for 42.22% of the total effect (Table 5, Figure 2).

**Table 5:** Mediating effect of error management climate between nursing informatics competency and information security attitude (*N* = 764).

Paths	Effect size	SE	95% Cl	*p*	Efficiency ratio (%)
Total effect					
Nursing informatics competency→information security attitude	0.495	0.030	[0.436,0.553]	<0.001	—
Direct effect					
Nursing informatics competency→information security attitude	0.286	0.029	[0.230,0.342]	<0.001	57.78
Indirect effect					
Nursing informatics competency→error management climate→information security attitude	0.209	0.020	[0.170,0.250]	<0.001	42.22

## Discussion

This study systematically described the current status of information security attitudes among clinical nurses, thoroughly explored the associations among nursing informatics competency, error management climate, and information security attitudes, and confirmed the mediating role of error management climate in this relationship. The research revealed three significant findings. Firstly, the information security attitudes of clinical nurses require further improvement. Secondly, there were significant associations among nursing informatics competency, error management climate, and information security attitudes. Finally, the study found that error management climate partially mediated the relationship between nursing informatics competency and information security attitudes.

### Current status of nurses’ information security attitude

The results of this study showed that the information security attitude score of clinical nurses were 94.00(82.00, 99.00), and the mean item score was 3.13(2.73, 3.30), which was at an upper-middle level. However, this was lower than the findings of Kang et al. (11) in their survey of 200 Korean nurses. The reason may be related to the relative lag in China in systematic nursing information security education, the construction of information security culture in medical institutions, and the implementation of related supervision. Among the six dimensions of information security attitude, the dimension of enhancing the sense of responsibility had the highest score, suggesting that clinical nurses attach more importance to the protection of patient privacy in various situations. The possible reason may be that nurses’ emphasis on patient privacy protection stems not only from professional ethics and legal norms, but also reflects their commitment to preserving patient dignity, building trust, and ensuring the quality of nursing care. In contrast, the dimensions of maintaining device stability and restricting information access had the lowest scores, and the possible reason is that nursing staff lack awareness and capability regarding ensuring the operation of information systems and managing access privileges. Therefore, nursing managers need to improve systems for access privileges and data security management, clarify principles for the allocation of privileges for different positions, and ensure that access to and use of patient information are consistent with job responsibility requirements. At the same time, by strengthening supervision and evaluation mechanisms, they should ensure that nurses’ data operation behaviors strictly follow standards, thereby reducing potential cybersecurity risks and providing a more solid security guarantee for medical services.

### Correlation among nursing informatics competency, error management climate, nurses’ information security attitude

This study found that clinical nurses’ nursing informatics competency was positively correlated with their information security attitude, that is, nurses with stronger nursing informatics competency often had more positive information security attitudes, and this finding was consistent with the conclusions of previous studies (12). This finding further corroborates the core logic of the Knowledge-Attitude-Practice (KAP) theory. When nurses’ nursing informatics competency is enhanced, it significantly boosts their self-efficacy in the field of information security, thereby fostering positive information security attitudes and behaviors (35, 36). The reason may be that nurses with higher levels of nursing informatics competency often receive more systematic education and training. Such training not only enhances their skills in information processing and application but also strengthens their awareness of responsibility for information security (37), ultimately forming a virtuous cycle of “knowledge-belief practice.” In clinical practice, nurses with a high level of nursing informatics competency can more sensitively identify potential information leakage risks (such as system vulnerabilities or non-standard operations), and can better understand and agree with the deeper value behind safety norms. This facilitates a cognitive and behavioral shift from “passive compliance” to “active implementation” (38). Therefore, nursing managers should establish normalized and periodic information security training systems within medical institutions, and organize nurses with outstanding performance in information security practice to share their practical experience, so as to promote clinical nurses’ nursing informatics competency and information security attitudes through diversified training approaches.

In addition, this study found a significant positive correlation between error management climate and information security attitude, indicating that creating a positive error management climate can improve nurses’ level of information security attitude. This result again confirms the findings of previous research (39), in which improvement in approaches to error handling made healthcare personnel more proactive in reporting adverse events, thereby holding a more positive attitude toward information security. A good error management climate reflects the organization’s way of accepting and handling errors, can influence nurses’ psychological safety, and promote their active engagement with issues related to information security, thereby improving overall information security attitudes (40). Specifically, when an organization creates a cultural climate that is inclusive and understanding toward errors, nurses are more willing to reflect and learn when facing errors, thereby strengthening their confidence in and emphasis on information security (41). Therefore, managers should take patient information security as the orientation to build a good error management climate. In this process, they should gain an in-depth understanding of nurses’ psychological needs and work pressure through effective communication, and integrate humanistic care with system construction, to create a management environment based on trust and support, thereby realizing the coordinated optimization of patient safety and nursing team development. In addition, by introducing a non-punitive adverse event reporting mechanism, protecting the privacy of reporters, and encouraging the active reporting of errors and hidden dangers, and by improving nurses’ information security awareness and error management capability through system analysis and experience sharing, it is possible to achieve the continuous improvement of nursing quality and the comprehensive safeguarding of patient safety.

This study also found that nursing informatics competency among clinical nurses has a positive impact on the error management climate, which means that clinical nurses with higher nursing informatics competency tend to have more standardized error management practices, which is consistent with the study by Chen Xu et al. (42). Studies have shown that nurses with solid nursing informatics competency can skillfully use informatization tools such as electronic nursing record systems and intelligent medication verification platforms to accurately capture potential error risks in key links such as execution of medical orders and patient identification (43). This practical experience of risk prediction weakens their avoidance psychology regarding errors, and further forms a behavioral tendency of active reporting and scientific handling. At the same time, nurses with higher nursing informatics competency can use informatization methods to summarize error data and analyze common problems, thereby providing objective support for team error review, promoting departments to actively carry out error review and improvement, and further shaping an error management climate of active prevention and continuous optimization (44). Therefore, managers should not only pay attention to the implementation of information training, but also pay attention to nurses’ ways of handling errors. By establishing an informatization error management platform to encourage active reporting and scientific review, a positive error management climate can be created.

### The mediating effect of error management climate on the relationship between nursing informatics competency and information security attitude

Our results showed that error management climate plays a partial mediating role in the relationship between clinical nurses’ nursing informatics competency and information security attitudes. Specifically, nursing informatics competency not only has a direct impact on information security attitudes, but can also indirectly influence information security attitudes by fostering a positive error management climate. This means that systematically improving nurses’ nursing informatics competency and enhancing the standardization of their error management can further promote the formation of positive and stable information security attitudes (45). Bandura’s social cognitive theory provides a reasonable explanation for this phenomenon: nursing informatics competency, as an important personal cognitive factor, promotes nurses to exhibit more standardized error management behaviors, which in turn helps to form an open and inclusive error management climate (environmental factor) (46). This supportive environment enhances nurses’ psychological safety, improves their self-efficacy, and provides continuous positive feedback, ultimately deepening nurses’ value recognition of and sense of responsibility for information security (attitudinal factor), thereby forming a virtuous cycle under the triadic reciprocal interaction. Therefore, nursing management is encouraged to implement targeted intervention measures to comprehensively improve clinical nurses’ nursing informatics competency and optimize the error management climate. Specific measures include improving the institutional framework for information security and error management, and focusing on cultivating nurses’ abilities in information risk identification, error response skills, and systematic thinking. In addition, differentiated training strategies should be implemented, with particular attention to the implementation and effectiveness of information security training in departments with high workload and frequent emergencies. At the same time, regular assessments or reward-and-punishment mechanisms should be adopted, and through positive incentives and appropriate constraints, promote the comprehensive improvement of nurses’ information security literacy.

### Limitations

This study has certain value in exploring the relationships nursing informatics competency among clinical nurses, error management climate, and information security attitude, but there are still several limitations. First, this study adopted a cross-sectional design, which cannot capture the dynamic changes of variables over time, resulting in the inability to establish causal relationships among the variables. Therefore, future studies should adopt longitudinal or experimental designs to explore the causal relationships among the model variables. Second, the use of convenience sampling may not fully reflect the actual situation of the clinical nurse population, which limits the generalizability of the findings. Thus, future research could expand the sample size and increase sample diversity. Third, data were collected using an online questionnaire survey, which may be subject to social desirability bias and selection bias, affecting the validity of the conclusions. Future studies may include some objective indicators to enhance the scientific rigor of the results. Fourth, the selection of variables in this study mainly focused on external factors such as age, hospital level, and departmental training, while relatively neglecting internal factors such as subjective initiative, self-efficacy, and professional values. Future research will further expand the range of variable selection and incorporate the above potential internal influencing factors into the analytical framework, so as to enhance the stability and explanatory power of the findings.

## Conclusion

This study systematically explored nursing informatics competency, error management climate, and information security attitudes among clinical nurses. It provides a theoretical basis for the interactions among these three factors and confirms that error management climate plays a partial mediating role between nursing informatics competency and information security attitudes. The findings support the triadic reciprocal determinism model of social cognitive theory, indicating that, in an environment of medical informatization, personal cognitive factors (nursing informatics competency), environmental factors (error management climate), and motivational factors (information security attitudes) jointly constitute a dynamic interactive system, thereby establishing self-efficacy and promoting nurses’ positive information security behaviors. Based on these findings, it is recommended that nursing managers foster a positive work environment through institutionalized interventions and continuous education, improve nurses’ information security attitudes and behaviors, and thereby enhance overall nursing quality.

## Data Availability

The raw data supporting the conclusions of this article will be made available by the authors, without undue reservation.

## Footnotes

1
https://www.wjx.cn
